# Coexpression Analysis of Transcriptome on AIDS and Other Human Disease Pathways by Canonical Correlation Analysis

**DOI:** 10.1155/2017/9163719

**Published:** 2017-06-14

**Authors:** Yahong Chen, Jinjin Yuan, Xianlin Han, Xiaolong Liu, Xiao Han, Hanhui Ye

**Affiliations:** ^1^The United Innovation of Mengchao Hepatobiliary Technology Key Laboratory of Fujian Province, Mengchao Hepatobiliary Hospital of Fujian Medical University, Fuzhou 350025, China; ^2^Infectious Diseases Hospital of Fuzhou, Fuzhou 350025, China; ^3^Department of General Surgery, Peking Union Medical College Hospital, Beijing, China; ^4^Biotechnology Research Institute, Chinese Academy of Agricultural Sciences, Beijing 100081, China

## Abstract

Acquired immune deficiency syndrome is a severe disease in humans caused by human immunodeficiency virus. Several human genes were characterized as host genetic factors that impact the processes of AIDS disease. Recent studies on AIDS patients revealed a series disease is complicating with AIDS. To resolve gene interaction between AIDS and complicating diseases, a canonical correlation analysis was used to identify the global correlation between AIDS and other disease pathway genes expression. The results showed that HLA-B, HLA-A, MH9, ZNED1, IRF1, TLR8, TSG101, NCOR2, and GML are the key AIDS-restricted genes highly correlated with other disease pathway genes. Furthermore, pathway genes in several diseases such as asthma, autoimmune thyroid disease, and malaria were globally correlated with ARGs. It suggests that these diseases are a high risk in AIDS patients as complicating diseases.

## 1. Introduction

Human immunodeficiency virus (HIV) causes a serious disease that affects people's health and lives. Millions of people have died from HIV infections in the 30 years since its identification. Over the past decades, a large number of studies have focused on every aspect of this virus, including virology, immunology, treatment, and genetics. An important problem related to AIDS complications was raised after the discovery of the HIV characteristics that severely damaged the lymphoid system.

Several diseases are associated with HIV infection and antiretroviral therapy. Tuberculosis is highly frequent in a large proportion of HIV infection cases in developing countries [1]. When research revealed that HIV-induced immune deficiency was the most common risk factor for cancer, HIV infection-related cancer became a complication of HIV infection [2]. HIV-associated sensory neuropathy is also a complication of HIV infection [3]. Recently, venous thrombosis has been described as a disease associated with HIV-positive patients [4]. Pulmonary arterial hypertension is a life-threatening complication of HIV infection [5]. A well-described complication of HIV and antiretroviral therapy is pancreatitis, which has exceedingly high rates in the HIV-positive population [6]. During antiretroviral therapy, classical Hodgkin lymphoma (HIV-cHL) and rhabdomyolysis are also important complications of HIV disease [7, 8]. One report showed that HIV patients frequently had neutropenia [9]. Generally, AIDS complications are involved in almost all important human diseases to the best of our knowledge.

Our understanding of genetic restriction factors targeting AIDS has been greatly improved by advances in genome research, such as sequencing of the whole human genome through physical and functional analyses. Many methods have been developed to study the underlying mechanisms of diseases on the whole genome level, such as genome-wide association studies, which can identify host genetic factors that affect HIV infection and the host restriction response. Nearly 40 AIDS restriction genes (ARGs) were identified from widely biological pathways such as the HIV entry receptor on lymphoid cells to oncogenes in human glioblastomas.

Many web-based databases, such as KEGG, have been established as tools to collect human disease pathway genes using genomic and molecular methods. For example, comparative transcriptome analysis can isolate marker genes that are highly differentially expressed in patients. Molecular biology has discovered several pathways that play major roles in human diseases, and hundreds of genes have been characterized as members of human disease pathways. Hence, human disease genes are available for the analysis of their effects on ARGs.

Expression correlation between genes based on a gene coexpression model can reveal the molecular mechanisms underlying gene regulation. For instance, mitochondrial pathways are coexpressed with muscle system pathway genes and neurodegenerative disease pathway genes [10]. The expression correlation between ARGs and other disease pathways could also explain the relationship between ARGs and AIDS complicating diseases. Canonical correlation analysis (CCA) is a powerful approach to detect coexpression between gene sets because it does not only determine correlations between two genes. For example, CCA can be used to perform a coexpression analysis of glioma pathway genes from glioblastoma transcriptomes.

In this study, we developed a CCA to determine coexpression between ARGs and other human disease pathway genes. We discussed the most significant coexpression patterns that could imply the susceptibility or sensitization to AIDS complicating diseases.

## 2. Methods

### 2.1. Datasets

Expression data on 20,000 human genes in human genome at about 4000 experiments was normalized to a human genome expression datasets hsa.v14-04.G19816-S5626 were downloaded from COPRESDB (http://coxpresdb.jp/). It includes many experiments not only related to HIV infection. Human disease pathway genes (br08402.keg updated 2016) were downloaded from KEGG (http://www.keg.jp); this dataset includes 68 typical disease pathways with key genes (Table 1). From published literature, we collected about 39 ARGs (see Table S1 available online at https://doi.org/10.1155/2017/9163719). From human genome expression datasets, two expression datasets were generated to include disease pathway genes and ARG expression data, respectively.

### 2.2. Canonical Correlation Analysis

Canonical correlation analysis is a statistical method which extracts statistically independent pair of canonical variables dependent on the correlation among two sets of original variables. The original variables are results of a linear combination of the canonical variables. In this study, expression of disease pathway genes in diverse conditions was described by vector *a* = (*a*_1_, *a*_2_,…, *a*_*m*_) and ARG expression by the vector *b* = (*b*_1_, *b*_2_,…, *b*_*m*_). The respective canonical variables *c* = (*c*_1_, *c*_2_,…, *c*_*m*_) and *d* = (*d*_1_, *d*_2_,…, *d*_*m*_) have canonical coefficients vectors *s* = (*s*_1_, *s*_2_,…, *s*_*m*_) and *s*′ = (*s*′_1_, *s*′_2_,…, *s*′_*m*_). *a* = *c*′s, and *b* = *d*′s′. The vector of eigenvalues was calculated as the magnitude of the correlation between pair of canonical variables. The variance covariance matrices were used to analyze the covariances between variables.

### 2.3. The Study Design and Software Tools

The R platform (http://www.rproject.org/) was used for canonical correlation analysis of expression data. After the canonical variables were produced, the top correlated canonical variables (*r* > 0.95) were identified to analysis the coexpressed individual genes. Two thresholds were set up to isolate correlated integrated disease pathways with *r*  values > 0.5 and standard deviations > 0.2. Web-based DAVID tool (http://david.abcc.ncifcrf.gov/) was used for functional annotations and enrichment analysis; we used *Homo sapiens* genome as background. The “KEGG_PATHWAY” was selected for disease pathway enrichment analysis. Other parameters were automatically generated from DAVID.

Functional annotations were generated, and enrichment analyses were performed for the metabolic pathway genes using the web-based DAVID tool (http://david.abcc.ncifcrf.gov/). For the pathway enrichment analyses, the “KEGG_PATHWAY” was selected. The pathways with a *P*  value < 0.01 were considered significant.

## 3. Results and Discussion

### 3.1. ARGs

Nearly 40 AIDS restriction genes (ARGs) have been considered as host genes that impact the progression of HIV infection from virus entry to the development of AIDS (Table S1). For example, PPIA, TSG101, TRIM5*α*, APOBEC3G, and CUL5 encode HIV-1 postentry cellular viral cofactors that have been described in recent research. PPIA plays a role in cyclosporin A-mediated immunosuppression as a member of the peptidyl-prolyl cis-trans isomerase (PPIase) family [11], which can interact with HIV viral proteins. Cell growth and differentiation are regulated by the interaction of TSG101 with stathmin [12]. TRIM5*α* is an E3 ubiquitin ligase that is involved in retroviral restriction [13]. Other ARGs are involved in many cellular processes, such as DEFB1, which has been implicated in cystic fibrosis pathogenesis [14], HLA-A, which is expressed in nearly all cells [15], CCL, which has been implicated in immunoregulatory and inflammatory processes [16], CXCR6, which is a chemokine (C-X-C motif) receptor [17], LY6D, which is a member of the lymphocyte antigen 6 complex [18], and APOBEC3B, which is a cytidine deaminase. However, these ARGs have been characterized only in the absence of AIDS-related complications. The relationship between ARGs and other human diseases is unknown.

### 3.2. The General CCA Results

The application of CCA to a transcriptome can identify coexpression between genes. Coexpression between individual genes or groups of genes can be identified based on the standard deviations of genes on canonical variables. Hence, we used two strategies to determine two types of correlation. First, the top (*r* > 0.95) canonical correlations with low standard deviations were isolated as coexpressed individual genes. Coexpression has been suggested to have less of an impact on whole disease pathways. The relationship between complicating diseases and AIDS is dependent on the roles of a few genes. Second, canonical correlations with high standard deviations (*s* > 0.2) and *r* values (>0.5) were selected as coexpressed between gene groups. This result indicates that coexpression has an effect on the whole disease pathway because more than 20% of the genes contribute to the canonical correlation. Most genes in disease pathways are involved in the cross talk between the complicating disease and AIDS.

### 3.3. Coexpressed ARGs and Human Disease Genes

As shown in Table 2, 21 top (*r* > 0.95) canonical correlations were determined between the ARGs and human disease pathway gene transcriptomes using the CCA approach. The canonical variables originated from disease pathways including HTLV-I infection, herpes simplex infection, Epstein-Barr virus infection, viral carcinogenesis, viral myocarditis, type I diabetes mellitus, graft-versus-host disease, autoimmune thyroid disease, allograft rejection, pathways in cancer, influenza A, proteoglycans in cancer, tuberculosis, transcriptional misregulation in cancer, Huntington's disease, toxoplasmosis, hepatitis B, measles, microRNAs in cancer, hepatitis C, and Alzheimer's disease. However, canonical variables could not delegate total ARG expression and human disease pathway gene expression because the standard deviations of the ARGs (Sa) and the human disease pathway genes (Sb) were less than 0.05, as shown in Table 2. Sa and Sb indicate that the canonical variables can explain less than 5% of the genes among the ARGs and human disease pathway genes. For example, HTLV-I infection pathway has 0.988 correlation factor with ARGs, and Wilks and Chisq indicate the statistic of correlation. But it has Sa < 0.01 and Sb < 0.05, which suggested that in whole pathway of HTLV-I infection, only a few genes were correlated with ARGs.

In Table 3, the genes with the highest correlation with the canonical variables from the ARGs and human disease pathway genes were collected to show the coexpression relationships.

#### 3.3.1. HLA-B and HLA-A

HLA-B and HLA-A are MHC class I molecules that strongly impact HIV-1 progression (Table 3) [19]. HLA polymorphisms are significantly correlated with the time to AIDS in HIV-infected individuals [20]. HLA-A and HLA-B are major AIDS restriction genes.

HLA-B and HLA-B were coexpressed with 3135 (HLA-G major histocompatibility complex, class I, G) and 3134 (HLA-F major histocompatibility complex, class I, F) genes shared by many disease pathways, including HTLV-I infection, herpes simplex infection, Epstein-Barr virus infection, viral carcinogenesis, viral myocarditis, type I diabetes mellitus, graft-versus-host disease, autoimmune thyroid disease, and allograft rejection (Table 3). As a marker of T cell activation, HLA-DR induction was associated with HTLV-I seropositivity [21]. HTLV-I infection leads to the induction of HLA [22]. Herpes simplex virus type 1 (HSV1) can upregulate HLA-G expression in human neurons after acute neuron infection [23], whereas HLA-G is the MHC class I molecule that is induced in B cells after Epstein-Barr virus transformation [24]. During viral carcinogenesis, HLA is abnormally expressed to enable cancer cells to escape from immune surveillance [25]. HLA-G polymorphisms and expression were suggested as diagnostic markers due to their involvement in breast carcinogenesis [26]. The increased occurrence of HLA antigens was shown to be associated with viral myocarditis [27]. The HLA complex has been reported to contribute to type 1 diabetes because HLA polymorphisms introduce genetic susceptibility to type 1 diabetes [28, 29]. There is evidence that HLA gene polymorphisms are potent risk factors for severe acute graft versus host diseases [30]. It was also shown that an HLA variant conferred a high risk for autoimmune thyroid disease [31]. Furthermore, allelic-induced abnormal expression of the HLA-G gene has been suggested to be associated with acute allograft rejection [32]. Generally, coexpression of the HLA genes indicates cross talk between HIV infection and other diseases, and this cross talk provides a potent mechanism for these diseases becoming AIDS complicating diseases.

#### 3.3.2. MYH9 and ZNRD1

MYH9 encodes a conventional nonmuscle myosin that is downregulated by HIV-1 pathogenesis [33]. As a zinc ribbon domain-containing protein, ZNRD1 plays a role in HIV-1 replication and AIDS progression [34]. A recent study found that ZNRD1 polymorphisms had a significant influence on AIDS progression [35]. ZNRD1 and MYH9 are major AIDS restriction genes.

Table 3 shows that MYH9 and ZNRD1 are coexpressed with different genes in disease pathways, such as 7428 (VHL; von Hippel-Lindau tumor suppressor) and 6772 (STAT1; signal transducer and activator of transcription 1) in cancer, 5781 (PTPN11; protein tyrosine phosphatase, nonreceptor type 11) and 5293 (PIK3CD; phosphatidylinositol-4,5-bisphosphate 3-kinase catalytic subunit delta) in proteoglycans in cancer, 5371 (PML; promyelocytic leukemia) and 51513 (ETV7; ETS variant 7) in transcriptional misregulation in cancer, 7143 (TNR; tenascin R) and 6655 (SOS2; SOS Ras/Rho guanine nucleotide exchange factor 2) in microRNAs in cancer, 2775 (GNAO1; G protein subunit alpha o1) and 4261 (CIITA; class II, major histocompatibility complex, transactivator) in toxoplasmosis, and 5111 (PCNA; proliferating cell nuclear antigen) and 356 (FASLG; Fas ligand) in hepatitis B.

VHL is a well-known tumor suppressor that is involved in hereditary cancer syndromes [36]. Inhibition of STAT1 can block cancer cell proliferation and invasion [37]. Overexpression of PTPN11 can enhance the progression of many proteoglycan cancers, such as liver cancer [38]. Inhibition of PIK3CD signaling can abrogate transitions in cancer cells [39]. Downregulation of PML, which is involved in the cell cycle, survival, and apoptosis, was identified in cancer cells [40]. ETV7 plays important roles in chromosomal translocations in human cancer [41]. Induction of SOS2 was demonstrated in cancer cells, such as triple negative breast cancer [42]. Toxoplasma gondii can downregulate CIITA to inhibit MHC class II expression [43]. The PCNA promoter can recruit the hepatitis B virus preS protein to become active [44]. Additionally, the Fas ligand gene can be induced by the hepatitis B virus X protein [45]. Thus, the coexpression of MYH9, ZNRD1, and other disease pathway genes suggests probable AIDS complicating diseases.

#### 3.3.3. IRF1 and ZNRD1

IRF1 is an interferon regulatory factor that can affect productive HIV infection and support natural resistance against HIV infection [46]. Hence, IRF1 is a well-studied human AIDS restriction gene.

As shown in Table 3, IRF1 and ZNRD1 are coexpressed with 356 (FASLG; Fas ligand) and 5609 (MAP2K7; mitogen-activated protein kinase 7) in the influenza A pathways. FASLG is described as a hepatitis B pathway gene and plays an important function in the influenza A virus pathway because influenza virus infection induces Fas ligand expression when the infected cells contact one another [47].

#### 3.3.4. TLR8 and ZNRD1

TLR8 is a human toll-like receptor that plays a role in signaling pathways that modulate the innate immune response to HIV infection and reduce HIV replication [48]. Genetic polymorphisms in TLR8 have been determined to be host cell factors associated with HIV status [49].

As shown in Table 3, TLR8 and ZNRD1 are coexpressed with 6772 (STAT1; signal transducer and activator of transcription 1) and 3587 (IL10RA; interleukin 10 receptor subunit alpha) in the tuberculosis pathways. STAT1 plays a role in a signaling pathway to control intracellular killing of phagocytosed *Mycobacterium tuberculosis* [50]. IL-10 is a key factor that mediates the immunopathogenesis of tuberculosis in combination with interferon gamma and adiponectin [51].

#### 3.3.5. TSG101 and ZNRD1

TSG101 interplays with the HIV-1 nucleocapsid to prohibit HIV from turning into a DNA-containing virus [52]. Therefore, TSG101 is a typical anti-HIV drug target and human AIDS restriction gene [53].

As shown in Table 3, TSG101 and ZNRD1 are coexpressed with 25981 (DNAH1; dynein axonemal heavy chain 1) and 55567 (DNAH3; dynein axonemal heavy chain 3) in the Huntington's disease pathways.

#### 3.3.6. NCOR2 and ZNRD1

NCOR2 is the RING finger ubiquitin ligase and nuclear corepressor that downregulates HIV-1 replication [54]. NCOR2 is a major AIDS restriction gene.

As shown in Table 3, NCOR2 and ZNRD1 are coexpressed with 356 (FASLG; Fas ligand) and 6772 (STAT1; signal transducer and activator of transcription 1) in the measles pathways. Fas ligand and STAT1 were previously described in other disease pathways. Moreover, the measles virus phosphoprotein can prevent STAT1 phosphorylation [55].

As shown in Table 3, NCOR2 and ZNRD1 are coexpressed with 4035 (LRP1; LDL receptor-related protein 1) and 9377 (COX5A; cytochrome c oxidase subunit 5A) in the Alzheimer's disease pathways. Recent studies showed that the LRP1 levels were reduced in Alzheimer's disease [56]. COX family genes can impair anxiety-like behavior in an Alzheimer's disease model [57].

#### 3.3.7. GML and ZNRD1

GML is the LY6 family member of the glycosylphosphatidylinositol- (GPI-) anchored proteins, which have conserved cysteine-rich domains with specific disulfide bonding patterns. LY6H was upregulated by HIV infection and suggested to play a role in innate immunity to HIV-1 via an interferon-like mechanism. Gene polymorphism analysis results support the hypothesis that GML is a susceptibility locus for HIV-1 infection [58].

GML and ZNRD1 are coexpressed with 6772 (STAT1; signal transducer and activator of transcription 1) and 6655 (SOS2; SOS Ras/Rho guanine nucleotide exchange factor 2) in the hepatitis C pathways. STAT1 plays an important role in hepatitis C infection in addition to cancer, tuberculosis, and measles. For example, hepatic STAT1 undergoes nuclear translocation during hepatitis C virus infection [59]. SOS2 has major functions in hepatitis C disease and cancer.

### 3.4. Coexpressed ARGs and Human Disease Pathways

As shown in Table 4, canonical correlations with large Sa, Sb (>0.2), and *r* values (>0.5) were selected to analyze coexpression between integral disease pathways and global ARGs. Unlike the top canonical correlations (*r* > 0.95) shown in Table 2, these canonical variables showed less correlation between each disease pathway and the ARG (Table 4). The maximum *r* was detected between the asthma pathways and ARGs (equal to 0.66). However, these canonical variables had larger standard deviations (at least 0.2) than the top canonical correlations described previously. In the maximum case, the Sa of the ARG correlated with the acute myeloid leukemia pathway was 0.37 and the Sb of the ARG correlated with the bacterial invasion of epithelial cells pathway was 0.47. Large standard deviations indicate that more ARGs or disease pathway genes are involved in the canonical variable. Additionally, these canonical correlations show a certain level of Pearson correlation, which determines coexpression. This result suggests that most of the genes in disease pathways are coexpressed with most of the ARGs at the whole pathway level. For example, renal cell carcinoma pathway showed coexpression with ARGs with *r* = 0.5, Sa = 0.26, and Sb = 0.21. It indicates that 26% ARG genes were expressed with 21% renal cell carcinoma pathway genes. Rather than find a single gene correlated with ARGs, CCA could identify a group of genes in certain pathways correlated with ARGs. This indicate more significant and higher potential than a single gene. These pathways include renal cell carcinoma, endometrial cancer, bladder cancer, acute myeloid leukemia, non-small-cell lung cancer, asthma, autoimmune thyroid disease, allograft rejection, graft-versus-host disease, primary immunodeficiency, nicotine addiction, type I diabetes mellitus, bacterial invasion of epithelial cells, pathogenic *Escherichia coli* infection, and malaria.

The coexpression identified from CCA is preferred to describe a general relationship since the transcriptome datasets include not only HIV-related experiments but also diverse biological experiments. Due to no limitation of samples in datasets, diseases in other human organs could be characterized as coexpressed pathway with AIDS. This provide a possibility to establish a precise experiment to determine this relationship.

Among these diseases, asthma is prevalent in populations with HIV infections because 20% of individuals infected with HIV have asthma in some clinical investigations [60]. There is evidence to support the hypothesis that asthma is one of the major causes of morbidity and mortality in HIV patients during antiretroviral therapy [61]. Recently, some studies have indicated that endometrial cancer has a favorable risk post-HIV infection [62]. One researcher found that some HIV drugs could inhibit in vitro bladder cancer migration and invasion [63]. In some clinical cases, HIV MDS transformed to acute myeloid leukemia [64]. A famous HIV-positive patient was healed of acute myeloid leukemia by allogeneic hematopoietic cell transplantation from a graft that carried the HIV-resistant CCR5 mutation [65]. The association between autoimmune thyroid disease and HIV infection has been suggested by several studies [66]. Although the HIV-related immunodeficiency state does not cause allograft rejection, anti-CD4 antibodies have considerable functions in the treatment of allograft rejection and the blockade of HIV infection [67, 68]. Recently, studies found that patients with HIV HAART-associated lipodystrophy syndrome had an increased risk of diabetes [69]. The impaired immune response to pneumococcal antigen pneumolysin due to HIV infection facilitates bacterial invasion [70]. Finally, HIV and AIDS are well-known subject diseases followed by malaria [71].

## 4. Conclusion

In this study, the correlation between ARGs and disease pathways genes expression were analyzed by CCA. The results showed that among ARGs, *HLA-B*, *HLA-A*, *MH9*, *ZNED1*, *IRF1*, *TLR8*, *TSG101*, *NCOR2*, and *GML* are the most significant genes correlated with other disease pathway genes. They are potential cross-links between AIDS and other diseases. Furthermore, gene pathways involved in several diseases such as asthma, autoimmune thyroid disease, and malaria were identified as an integrated pathway correlated with integrated ARGs. It suggests the risk of these diseases as AIDS complicating disease.

## Supplementary Material

Table 1 HIV host genetic factor genes.

## Figures and Tables

**Table 1:** Human disease pathway in KEGG.

Classes	ID	Pathways
Cancers: overview	5200	Pathways in cancer
	5202	Transcriptional misregulation in cancer
	5203	Viral carcinogenesis
	5204	Chemical carcinogenesis
	5205	Proteoglycans in cancer
	5206	MicroRNAs in cancer

Cancers: specific types	5210	Colorectal cancer
	5211	Renal cell carcinoma
	5212	Pancreatic cancer
	5213	Endometrial cancer
	5214	Glioma
	5215	Prostate cancer
	5216	Thyroid cancer
	5217	Basal cell carcinoma
	5218	Melanoma
	5219	Bladder cancer
	5220	Chronic myeloid leukemia
	5221	Acute myeloid leukemia
	5222	Small cell lung cancer
	5223	Non-small-cell lung cancer

Immune diseases	5310	Asthma
	5320	Autoimmune thyroid disease
	5321	Inflammatory bowel disease (IBD)
	5322	Systemic lupus erythematosus
	5323	Rheumatoid arthritis
	5330	Allograft rejection
	5332	Graft-versus-host disease
	5340	Primary immunodeficiency

Neurodegenerative diseases	5010	Alzheimer's disease
	5012	Parkinson's disease
	5014	Amyotrophic lateral sclerosis (ALS)
	5016	Huntington's disease
	5020	Prion diseases

Substance dependence	5030	Cocaine addiction
	5031	Amphetamine addiction
	5032	Morphine addiction
	5033	Nicotine addiction
	5034	Alcoholism

Cardiovascular diseases	5410	Hypertrophic cardiomyopathy (HCM)
	5412	Arrhythmogenic right ventricular cardiomyopathy (ARVC)
	5414	Dilated cardiomyopathy
	5416	Viral myocarditis

Endocrine and metabolic diseases	4930	Type II diabetes mellitus
	4940	Type I diabetes mellitus
	4950	Maturity onset diabetes of the young

Infectious diseases: bacterial	5100	Bacterial invasion of epithelial cells
	5110	*Vibrio cholerae* infection
	5120	Epithelial cell signaling in *Helicobacter pylori* infection
	5130	Pathogenic *Escherichia coli* infection
	5131	Shigellosis
	5132	Salmonella infection
	5133	Pertussis
	5134	Legionellosis
	5150	*Staphylococcus aureus* infection
	5152	Tuberculosis

Infectious diseases: viral	5160	Hepatitis C
	5161	Hepatitis B
	5162	Measles
	5164	Influenza A
	5166	HTLV-I infection
	5168	Herpes simplex infection
	5169	Epstein-Barr virus infection

Infectious diseases: parasitic	5140	Leishmaniasis
	5142	Chagas disease (American trypanosomiasis)
	5143	African trypanosomiasis
	5144	Malaria
	5145	Toxoplasmosis
	5146	Amoebiasis

**Table 2:** Typical disease pathways have high coexpression with ARGs. *r* is the correlation factor; Sa, standard deviation of canonical variables in a (ARGs); Sb, standard deviation of canonical variables in b (disease pathways); Wilks, Wilks' lambda (likelihood ratio) statistic; and Chisq, Bartlett's approximate chi-squared statistic for H(k)0 with Lawley's modification.

Pathway ID	Pathways	*r*	Sa	Sb	Wilks	Chisq
5166	HTLV-I infection	0.988	0.0064	0.0119	5.56*E* − 14	129868.5
5168	Herpes simplex infection	0.987	0.0064	0.0112	1.22*E* − 11	107961
5169	Epstein-Barr virus infection	0.987	0.0065	0.0120	4.46*E* − 12	112040.6
5203	Viral carcinogenesis	0.987	0.0068	0.0126	1.05*E* − 11	108466.7
5416	Viral myocarditis	0.983	0.0073	0.0130	1.24*E* − 07	69145.74
4940	Type I diabetes mellitus	0.982	0.0078	0.0145	8.94*E* − 06	50709.06
5332	Graft-versus-host disease	0.981	0.0080	0.0141	2.50*E* − 05	46246.41
5320	Autoimmune thyroid disease	0.981	0.0083	0.0149	2.52*E* − 05	46159.16
5330	Allograft rejection	0.980	0.0081	0.0146	6.08*E* − 05	42385.9
5200	Pathways in cancer	0.972	0.0091	0.0103	1.39*E* − 14	134581
5164	Influenza A	0.964	0.0079	0.0086	7.45*E* − 11	100289.5
5205	Proteoglycans in cancer	0.964	0.0116	0.0088	4.19*E* − 12	111889.7
5152	Tuberculosis	0.960	0.0083	0.0088	6.84*E* − 11	100606.8
5202	Transcriptional misregulation in cancer	0.959	0.0107	0.0097	9.35*E* − 11	99265.29
5016	Huntington's disease	0.958	0.0209	0.0162	1.09*E* − 09	88602.38
5145	Toxoplasmosis	0.958	0.0093	0.0089	8.41*E* − 09	80437.34
5161	Hepatitis B	0.957	0.0117	0.0101	6.25*E* − 10	91320.55
5162	Measles	0.956	0.0098	0.0105	1.27*E* − 09	88462.56
5206	MicroRNAs in cancer	0.956	0.0131	0.0093	4.63*E* − 10	92549.92
5160	Hepatitis C	0.952	0.0129	0.0111	3.99*E* − 09	83502.52
5010	Alzheimer's disease	0.951	0.0192	0.0153	1.18*E* − 09	88451.59

**Table 3:** AIDS-restricted genes have high coexpression with other diseases.

ID	Pathways	ARGs	Pathway genes
5166	HTLV-I infection	HLA-B, HLA-A	3135, 3134
5168	Herpes simplex infection	HLA-B, HLA-A	3135, 3134
5169	Epstein-Barr virus infection	HLA-B, HLA-A	3135, 3134
5203	Viral carcinogenesis	HLA-B, HLA-A	3135, 3134
5416	Viral myocarditis	HLA-B, HLA-A	3135, 3134
4940	Type I diabetes mellitus	HLA-B, HLA-A	3135, 3134
5332	Graft-versus-host disease	HLA-B, HLA-A	3135, 3134
5320	Autoimmune thyroid disease	HLA-B, HLA-A	3135, 3134
5330	Allograft rejection	HLA-B, HLA-A	3135, 3134
5200	Pathways in cancer	MYH9, ZNRD1	7428, 6772
5164	Influenza A	IRF1, ZNRD1	356, 5609
5205	Proteoglycans in cancer	MYH9, ZNRD1	5781, 5293
5152	Tuberculosis	TLR8, ZNRD1	6772, 3587
5202	Transcriptional misregulation in cancer	ZNRD1, MYH9	5371, 51513
5016	Huntington's disease	ZNRD1, TSG101	25981, 55567
5145	Toxoplasmosis	MYH9, ZNRD1	2775, 4261
5161	Hepatitis B	MYH9, ZNRD1	5111, 356
5162	Measles	NCOR2, ZNRD1	356, 6772
5206	MicroRNAs in cancer	MYH9, ZNRD1	7143, 6655
5160	Hepatitis C	ZNRD1, GML	6772, 6655
5010	Alzheimer's disease	NCOR2, ZNRD1	4035, 9377

**Table 4:** Canonical correlation between integrated disease pathway genes and ARGs.

ID	Pathways	Pair ID	Sa	Sb	*r*
5211	Renal cell carcinoma	14	0.26	0.21	0.5
5213	Endometrial cancer	12	0.26	0.21	0.51
5219	Bladder cancer	11	0.29	0.22	0.5
5221	Acute myeloid leukemia	12	0.37	0.21	0.52
5223	Non-small-cell lung cancer	8	0.23	0.21	0.62
5310	Asthma	4	0.22	0.42	0.66
5320	Autoimmune thyroid disease	7	0.27	0.4	0.57
5330	Allograft rejection	7	0.31	0.26	0.52
5332	Graft-versus-host disease	4	0.21	0.23	0.72
5340	Primary immunodeficiency	8	0.22	0.24	0.55
5033	Nicotine addiction	3	0.35	0.41	0.58
4940	Type I diabetes mellitus	6	0.24	0.24	0.64
5100	Bacterial invasion of epithelial cells	4	0.21	0.49	0.62
5130	Pathogenic *Escherichia coli* infection	12	0.25	0.21	0.58
5144	Malaria	8	0.24	0.21	0.54
